# Palliative psychiatry through the eyes of psychiatric nurses in a country where palliative psychiatry has not yet emerged: A qualitative study

**DOI:** 10.1017/S1478951526101679

**Published:** 2026-03-26

**Authors:** Emel Erdeniz Güreş, Azize Atlı Özbaş

**Affiliations:** 1 Research Assistant, Faculty of Health Sciences, Department of Nursing, Uskudar University, Istanbul, Türkiye; 2 PhD Candidate, Department of Psychiatric Nursing, Hacettepe University, Ankara, Türkiye; 3 Associate Professor, Department of Psychiatric Nursing, Hacettepe University, Ankara, Türkiye

**Keywords:** Palliative care, palliative psychiatry, psychiatric illness, psychiatric nursing, nursing

## Abstract

**Objectives:**

Palliative psychiatry is an approach that aims to prevent and/or alleviate suffering and improve the quality of life of patients and their families through timely assessment and treatment when faced with physical, psychological, social, and spiritual problems associated with serious life-threatening mental illness. However, the need for psychiatric palliative care for individuals with serious mental illness has remained in the background and only became possible in the early 21st century. Therefore, it is essential that nurses, one of the most important actors of patient-centered care, assume the responsibility of providing palliative psychiatric care as a requirement of their patient advocacy role. The aim of this study was to determine the perception of palliative psychiatric care by psychiatric nurses in a country where palliative psychiatry has not yet emerged.

**Methods:**

A study in qualitative descriptive design with semi-structured interviews. Fifteen psychiatric nurses participated in individual interviews. The data were analyzed using thematic analysis.

**Results:**

Four themes (Perception of palliative psychiatric care, Palliative psychiatric care practices, Barriers to palliative psychiatric care, and Recommendations for providing palliative psychiatric care) and 14 sub-themes were identified.

**Significance of results:**

Psychiatric nurses are not familiar with the concept of palliative psychiatric care and associate it with holistic and individualized care. Nurses stated that palliative psychiatric care targets serious psychiatric disorders, treatment-resistant conditions, comorbidities and end-of-life care. In order to overcome barriers to palliative psychiatric care, suggestions were made for reorganizing the health system, establishing palliative care centers, training professionals in this field, and efforts to combat stigma.

## Introduction

Psychiatric patients are a disadvantaged group in terms of access to health services, although they are more likely to have physical health problems. Therefore, difficult-to-treat physical illnesses are more common in this group. Psychiatric patients are at risk of dying at an earlier age than healthy individuals due to poor quality of life, late diagnosis, difficulties in adherence to treatment and lack of help-seeking behaviors (Copeli and Cohen 2020). For these reasons, this group has inadequate access to psychiatric care, specialized treatment, health screenings, and early diagnostic assessments. Clinical and personal recovery models are used in the treatment of mental illness. Clinical recovery aims for symptoms reduction, absence of relapse, and improved psychosocial functioning, while personal recovery aims for personal growth, independence, and a meaningful life (Davidson and Guy 2012; Jaeger and Hoff 2012; Drake and Whitley 2014). Palliative psychiatry embodies both of these types of recovery, supporting the individual in achieving their individual life goals through self-determination, autonomy, dignity, and acceptance. However, palliative psychiatry aims to function in the illness process in conjunction with other approaches to prevention, cure, rehabilitation, or recovery. Therefore, in recent years, it has been suggested that palliative care approaches should also be considered for people diagnosed with severe mental illness (Cooper and Cooper 2018; Trachsel et al. 2019). Palliative psychiatric care is based on the recognition that curative treatment may not always be possible, but even then the patient and family may benefit from interventions aimed at symptom control and quality of life and suggests that the focus should be on improving quality of life rather than treatment in treatment-resistant cases where no progress can be made despite various treatment efforts and drug trials (Decorte et al. 2020; Eisenmann et al. 2020). This approach does not recommend palliative care as a substitute for treatment interventions, but rather encourages its use in combination with rehabilitation and recovery-oriented treatments (Trachsel et al. 2019; Zumstein and Riese 2020). The palliative care approach aims for each patient to achieve “a satisfying lifestyle despite the limitations caused by the disease” and for each individual to develop “new meaning and purpose” beyond the devastating effects of mental illness (Decorte et al. 2020).

Emerging in the 1950s as an extension of the hospice concept, palliative care has traditionally focused on cancer patients but now includes individuals with neurological disorders and chronic illnesses like AIDS and multiple sclerosis (Brooksbank 2009). However, psychiatric patients are also considered to be an important population that will benefit from palliative care practices. Evidence suggests that palliative psychiatry improves the quality of life of patients and their families in conditions such as schizophrenia, persistent eating disorders, recurrent suicide attempts, severe depression, and dementia (Elie et al. 2018; Decorte et al. 2020; Gotanda et al. 2022; Carter and Mizelle 2023; George 2023; Westermair and Trachsel 2023).

Palliative care is a relatively new area of interest in Turkey. Historically, efforts to develop palliative care began in the late 2000s, and in 2008, the Turkish Ministry of Health, Department of Cancer Control launched the National Palliative Care Program. In 2009, the Ministry of Health launched the 5-year National Cancer Control Program focusing on cancer patients and their relatives. The Pallia-Turk project, one of the important steps of the program, aims to create a palliative care model by analyzing cancer data and human resources, to establish at least three pilot palliative care centers, to create trained and experienced professional teams in the field of palliative care (implementation and management), and to facilitate the availability and usability of opioids. Within the scope of legal regulation for palliative care, the “Directive on the Implementation Procedures and Principles of Palliative Care Services” entered into force in 2014 (Gültekin et al. 2010; Kıvanç 2017). Palliative care has attracted considerable interest among scientists and nurses and a considerable number of studies have been conducted nationally (Gültekin et al. 2010; Kıvanç 2017). However, these studies have mostly focused on cancer patients and symptom control (Gültekin et al. 2010; Kıvanç 2017). When we look at the concept of palliative care from a broader perspective, it is stated that it should also cover physical chronic and serious mental illnesses other than cancer, and that individuals and their families should be addressed physically, mentally, and psychosocially from the onset of the disease.

Nurses, as innovators and leaders, are in an important position in areas such as medication management, education, symptom management support, and psychosocial support, in addition to traditional medical management, for individuals with serious mental illness due to their holistic perspective and patient-centered focus (Scott and Scott 2021). The important role of nurses in the care of psychiatric patients will require a holistic approach to palliative care services for people with severe mental illness. At this point, it is very important that nurses have this understanding and advocate for palliative care practices that their patients need. This study fills a gap in both the national and international arena and explores the views and opinions of nurses working in psychiatric services in relation to psychiatric palliative care.

## Methods

### Study design and setting

A descriptive qualitative study was conducted to determine psychiatric nurses’ perception of psychiatric palliative care. This approach was adopted because it is an appropriate method that can be used in cases where there is limited information on the subject under investigation, the richness of the data collected and the opportunity to obtain the views of psychiatric nurses on the perception of psychiatric care in depth (Doyle et al. 2020).

This study was reported according to the Consolidated Criteria for Qualitative Research (COREQ) checklist (Tong et al. 2007).

### Participants

The nurses working in psychiatric clinics included in the study sample were reached through the Psychiatric Nursing Association. The announcement of the study was published on PHD social media, and volunteer participants were contacted by the researchers. Online interviews were conducted with the participants on the specified day and time. The study’s sample size meets the criteria of 5–25 participants recommended for qualitative research (Polit and Beck 2021). The researchers concluded that data saturation was reached at the 15th participant, and 15 participants constituted the sample. Participants were not withdrawn from the study at any stage. During the online interviews, there was no one else in the environment except the interviewer and the participants. Interviews were conducted on a one-to-one basis. The interviewer and the participants did not know each other beforehand.

### Data collection

Data were collected online, through in-depth interviews using a semi-structured questionnaire between 05/06/2023 and 10/01/2024, following ethics committee approval. A pilot study with 3 participants confirmed the interview form’s validity; thus, they were included in the sample. Subsequently, individual interviews were conducted online at convenient times for the nurses, with only the researcher and participant present. Audio recordings were taken during each session.

In qualitative research, data saturation determines sample adequacy (Gentles et al. 2015). Therefore, in this study, data saturation was used to determine the sample size. We used some criteria to determine data saturation. The first of these criteria was the repetition of coding patterns. The second was information based on data saturation in the literature. In qualitative research, determining sample size is context-specific and largely depends on the scientific paradigm of the study. The literature suggests that studies utilizing a single phenomenon can potentially reveal highly significant and novel areas. Studies with a single phenomenon can reveal significant findings, often achieving saturation with around 12 participants in homogeneous groups (Boddy 2016). This study, codes repeated after the 12th interview. Three consecutive interviews were then conducted to ensure that no new content was received. Three more interviews confirmed no new content. Data collection ended after the 15th interview, assuming saturation was reached.

Author 2 conducted interviews after obtaining participants’ consent. The interviews were audio recorded and lasted an average of 40–52 min, and transcribed within a week.

All interviews were in Turkish. Translation to English occurred after content analysis by a skilled translator and was professionally edited. Transcripts were sent to 3 participants to obtain thematic approval.

### Data analysis

This study employed content analysis to explore participants’ experiences, beliefs, and thoughts (Kyngäs 2020). Participants’ voice recordings were transcribed and read by the researcher (Author 2). Authors 1 and 2 carried out the analysis independently of each other using a systematic approach: First, they independently read the texts repeatedly to obtain meaningful insights. In the next stage, key statements directly related to the phenomenon were selected and important units of meaning were identified and coded according to explicit and implicit meanings. Third, the resulting codes were categorized and grouped to reveal common concepts or themes; each researcher independently developed themes and sub-themes by identifying patterns within the codes. Fourth, the researchers compared the codes, sub-themes, and themes. They then revisited the relevant texts to refine the themes and sub-themes. Finally, the 2 researchers agreed on the sub-themes and themes and interpreted them in a comprehensive report (Kyngäs 2020). Finally, 4 themes and 14 sub-themes were identified.

### Ethical considerations

On 11 September 2020, the Non Interventional Clinical Research Ethics Committee of XXX approved this study (ref nr.: 61351342/September 2022–39). The purpose of the study was explained to the participants, and they were informed that their data would remain confidential. All provided informed consent to participate in the study.

### Rigor/trustworthiness

The study aimed to ensure reliability through criteria such as transferability, credibility, confirmability, and trustworthiness (Ahmed 2024). To achieve this, researchers thoroughly described the study setting, participants, sampling method, demographics, inclusion/exclusion criteria, interview procedures, and interview guide in the methodology section.

The study’s rigor was ensured by applying readability, transferability, reliability, and relevance criteria as suggested by Guba et al. (Guba and Lincoln 1989). Participants from eleven institutions contributed insights on palliative psychiatric care. The data were used and transcribed as direct citations from semi-structured interviews without commentary. Themes and subthemes were identified and grouped to ensure credibility and reliability. Details on inclusion/exclusion criteria, participant characteristics, contexts, data collection, and analysis were provided (Carpenter et al. 2011). To avoid confirmation bias, researchers cross-checked quotations and finalized themes collaboratively.

Reliability was ensured following Brinkmann and Kvale’s (2015) guidelines (Brinkmann and Kvale 2015). Researcher triangulation was applied, involving multiple researchers in question formulation, data collection, analysis, and interpretation. Consistency was maintained using a standardized semi-structured interview guide refined through a pilot study. Researchers rehearsed interviews beforehand, and participants provided informed consent, ensuring the right to withdraw, thereby enhancing reliability.

### Research team and reflexivity

Reflexivity was maintained by the research team through the analysis and writing by recording, discussing and challenging established assumptions. The research team consisted of two nurse academics specialized in the field of nursing. Researcher (Author 1) was a woman, a PhD holder in psychiatric nursing. Researcher (Author 2) was a woman PhD student in psychiatric nursing and a research assistant at a university. The researchers were trained in qualitative research methods and had qualitative research experience.

## Results

The mean age of the participants was 32.13 ± 5.36 years, and their experience in mental health services was 8.31 ± 5.56 years. The participants' characteristics are presented in Table 1.

**Table 1.** Characteristics of interview participants

Participant	Age	Gender	Professional experience (years)	Education
N1	28	Female	6 year	Undergraduate
N2	31	Female	8 year	Master’s degree
N3	32	Man	9 year	Master’s degree
N4	26	Female	1 year	Master’s degree
N5	42	Female	18 year	Undergraduate
N6	25	Female	1 year	Undergraduate
N7	30	Female	6 year	Master’s degree
N8	26	Female	1 year	Master’s degree
K9	38	Female	15 year	Undergraduate
N10	34	Female	10 year	Undergraduate
N11	27	Female	2 year	Master’s degree
N12	32	Female	7 year	Master’s degree
N13	28	Female	5 year	Master’s degree
N14	30	Female	6 year	Master’s degree
N15	30	Female	3 year	Master’s degree

The findings of this study, which was conducted to examine the views of nurses working in psychiatric services on palliative psychiatric care, were analyzed under four themes. The participants' perceptions of palliative psychiatric care are presented in Table 2.

**Table 2.** Participants’ Perception of Palliative Psychiatric Care

Theme	Sub-theme
**Perception of Palliative Psychiatric Care**	Lack of Knowledge in Palliative Psychiatry
	Aim of Psychiatric Palliative Care
	Target Populations For Psychiatric Palliative Care
**Palliative Psychiatric Care Practices**	Physical Care
	Psychosocial Care
	End of Life Care
**Barriers to Palliative Psychiatric Care**	Stigmatization/ Negative Discrimination
	Lack of Competence of The Health Team
	Medicine-Focused Care and Treatment Paradigm
	System-related Barriers
**Suggestions for Providing Palliative Psychiatric Care**	Palliative Care Center
	Training of Health Professionals
	Combating Stigma
	Regulation in Healthcare System

### Theme 1: Perception of palliative psychiatric care

Participants’ perception of palliative psychiatric care is analyzed in three sub-themes: lack of knowledge in palliative psychiatry, aim of palliative psychiatric care, target populations for palliative psychiatric care.

### Sub-theme 1: Lack of knowledge in palliative psychiatry

Participants said that they had never heard of palliative psychiatry before. In order to define palliative psychiatry, they drew on their perceptions of palliative care and their experiences of psychiatry.

In that way, I don’t know whether it is palliative care or not, but in my opinion, I think their adaptation to treatment, their regular use of medication…. I don’t know, Actually that I said. (Participant 8)

I first thought of palliative care practices directly as elderly patients, because elderly and seriously ill patients come in, and they have serious care needs. I thought of it as meeting them.” (Participant 4)

As palliative care in psychiatry, we have a bath day twice a day. The things we do as palliative care are to make the patient take a bath, to try to make the patient take a bath, to make him/her do it himself/herself if he/she does not do it, to take care of his/her nails, to take care of his/her hair, to take care of his/her beard. There is a daily body care in palliative care. We do not do that, we make them do it themselves. We do all of these things, You know… as palliative care. (Participant 6

### Sub-theme 2: Aim of psychiatric palliative care

Participants identified the aims of palliative psychiatric care as managing acute conditions, symptom control, addressing physical care needs, providing psychosocial support, ensuring treatment adherence, involving family in care, and reducing stigmatization.

In palliative psychiatric care, first of all, the management of the patient in the acute period, primary management should be provided. The patient will not have the ability to evaluate reality. He will also have psychiatric findings. They will have active hallucinations and delusions. (Participant 14)

Because people with mental illness experience the most stigmatization in the family, they are generally uncared for, unwanted patients. Their needs are not met and they have problems. They lack support from the society and the family. We should work on this issue. (Participant 4)

### Sub-theme 3: Target populations for palliative psychiatric care

Participants identified individuals with severe psychiatric disorders, failed disease management, comorbidities and end-of-life care needs as requiring palliative psychiatric care.

I mean, I have a patient with severe schizophrenia and he cannot be controlled in any way. He has a lot of relapses…. It is very important for the patient to receive palliative psychiatric care to increase their well-being and to minimize visual or auditory hallucinations or delusions. (Participant 11)

… Unfortunately, patients mostly lose their lives at home. They can experience traumatic things especially during the period. For example, as a result of a fire in the house, they can lose their lives by burning because they can no longer continue to function. (Participant 9)

### Theme 2: Palliative psychiatric care practices

Palliative psychiatric care practices are analyzed in three sub-themes: physical care, psychosocial care and end-of-life care practices.

### Sub-theme 1: Physical care

Participants included self-care support, fluid support, medication support, sleep hygiene and exercise in the scope of physical care.

First of all, the care needs of these patients, first of all, their medication is the most basic thing, … . Apart from that, when I think about the care of these patients, personal care, control of their medications, spiritual care are important in this sense. (Participant 11)

After they are discharged, we explain these to the patients like a continuous advice, we do it in the form of small trainings, such as walking for half an hour, drinking two liters of water, eating these foods, salt intake is important in the medication you use, you should pay attention to your sleep, in order to ensure that they live their lives more comfortably and with better quality. (Participant 4)

### Sub-theme 2: Psychosocial care

Participants included psychosocial care including psychoeducation, symptom control, socialization, promotion of independence, individualized interventions and therapies.

Maybe it could be motivating and helping to make adjustments in your social life, in your life outside the hospital to reduce or prevent repeated hospitalizations. Maybe directing them to practices in community health centers. … (Participant 13)

### Sub-theme 3: End of life care

Participants emphasized that patients do not receive end-of-life care and that this care requires collaboration with other health professionals.

Or they are not taken to a health institution, saying that they were already sick. You know, it can be looked at with the view that he will die anyway. Or, for example, let’s say the patient applies to the emergency room. In a situation that is close to the end of life, even when he/she applies to the emergency room, he/she can be immediately hospitalized in the psychiatric ward by being called a psychiatric patient. (Participant 9)

What we call palliative care has always been defined as improving the quality of life of patients at the end of life and relieving the pain of the rest. … So we saw it as a comfortable death. The vast majority of patients who are registered in community mental health centres, for example, have committed suicide or have left the institution as ex-patients. You know that these patients are at least somewhat better, so this ending isn’t called a happy ending, but I still don’t know how to say it. …” (Katılımcı 2)

### Theme 3: Barriers to palliative psychiatric care

Barriers to palliative psychiatric care fall into four sub-themes: stigmatization, inadequate competence of healthcare teams, a medical-centered care paradigm, and system-related barriers.

### Sub-theme 1: Stigmatization/negative discrimination

Participants reported stigmatization by family, healthcare professionals, internalized stigma, and diagnosis-specific discrimination. While psychiatric patients face general discrimination, older patients needing physical care and those with additional diagnoses, such as substance abuse or personality disorders, experience heightened discrimination.

Social support is inadequate as patients’ family ties are broken. For example, while the family of a diabetic patient provides more support in the form of let’s check their blood sugar, let’s pay attention to their food, people with mental illness experience the most stigmatization in the family. In general, they can be stigmatized as uncared for, unwanted, the patient who does bad things… Patients’ needs are not met. They do not receive enough support from both the community and the family. (Participant 4)

Because psychiatric patients still experience stigmatization whether they are doctors, nurses or medical secretaries. In fact, it exists for every professional group. (Participant 15)

The people working in these psychiatric units, the nurses, are mostly nurses who have reached a certain age and are taken to rest, who have no theoretical knowledge about the approach to psychiatric patients and psychiatry, who are generally older and generally have secondary education degrees. (Participant 7)

For example, it is very difficult to give perineal care, bed bathing, etc. in elderly patients with major depression. They have no motivation. It is also very difficult to have to do this care by applying physical force. It is also difficult for me to try to communicate with the elderly group. I feel that I am not understood by the elderly. It is also very difficult to convince them. (Participant 13)

I have difficulty in maintaining the boundary in communication with substance patients because they have a more you and me communication. This makes me a little uncomfortable. (Participant 13)

### Sub-theme 2: Lack of competence of the health team

Participants reported a lack of awareness and training in palliative psychiatric care among healthcare teams. Barriers included insufficient multidisciplinary collaboration, physician hierarchy, and communication issues. Additionally, participants lacked experience managing physical comorbidities alongside psychiatric illnesses.

For example, we had a patient… She had been schizophrenic for many years and was already a very destroyed patient. Then the patient’s condition deteriorated more… At this point ileus had developed in the patient. This was recognised late. At that time, for example, there was a nurse have intensive care experience. The patient becomes hypoxic and distended. They went to a lot of trouble. You know, sending blood gases, other interventions. You know, to make the patient breathe a bit more comfortably, to calm him down a bit more, to tidy him up, you know, even to recognise process… They did a lot of interventions there. You know, because it is psychiatry clinic, the team has no experience. You know, the nurse experienced intensive care gave some instructions. But the nurse who came the next day has not any experience about intensive care, for example, had difficulties… (Participant 9)

For example, for nurses like me who opened their eyes in psychiatry: What is patient care first and foremost? Physically, how do you care for a patient? How do you do it, even if it is just a bandage? Yes, we learn in school, but what we learn in school is actually not very effective… . For people who started directly in psychiatry setting, maybe a few months of rotation would be enough. Even if there was no rotation, there could have been training. As I said, how can there be physical patient care? At least about the things we often encounter according to our own patient profile. (Participant 13)

When we convey an observation about the patient to the clinical chief, we feel that we are not given much importance. When you perform an intervention on the patient, even if it benefits the patient, we see that the intervention is not included in the whole treatment. (Participant 13)

The emphasis is only on medication. But we lack a lot in terms of care. There are still nurses who see nursing only as a drug treatment practitioner. …Unfortunately, they do not know what it means to provide psychosocial and spiritual care to the patient (Participant 8)

### Sub-theme 3: Medicine-focused care and treatment paradigm

Participants reported that clinics prioritized medical treatment, neglected holistic care, undervalued quality of life, and disregarded care services. This approach persists due to administrative detachment from psychiatry, physician-dominated culture, and lack of recognition of nurses’ independent roles.

Therefore, we should be aware that we need to look more holistically. Doctors are more interested in the medical condition, diagnosis, medication, psychiatric condition. (Participant 4)

We are not supported by the managers of the institution in palliative care practices. There is a point of view that ‘In psychiatric clinics, if the patient does not harm himself or does not escape from the ward, it is the best ward. (Participant 15)

### Sub-theme 4: System-based barriers

Participants identified system-related barriers to palliative psychiatric care, including inadequate preventive healthcare, limited resources, service discontinuity, lack of standardization, absence of palliative care centers, and insufficient legal regulations. Additionally, the non-standardization of the services provided between institutions and the inability of patients to receive a service that includes follow-up, follow-up and care after discharge were also among the statements of the participants.

The number of nurses is low, physical conditions are inadequate and the number of patients is too high. For example, there should be two patients in each room. We have rooms for 5-6 patients. We do the treatment by queuing the patient. The patient comes and has to queue. This does not fit the individualised care model. There is not even a meeting room to talk to the patient about their treatment. (Participant 12)

Our institution is very deficient in this sense. There is no rehabilitation service at all. … In this sense, we need to gain an institutional perspective. In other words, when we identify the needs and convey them to our administrators, they respond that “these are the possibilities. (Participant 12)

For example, there is a lot of medication non-compliance after patients are discharged. In order to better follow up on these, community mental health centres need to be improved in terms of both quantity and quality. And there needs to be a standard. (Participant 7)

When the patient’s condition worsens due to both medications and other comorbid diseases, who will serve the patient or how will they be followed up? This is ambiguous. (Participant 9)

### Theme 4: Suggestions for providing palliative psychiatric care

Participants’ suggestions for palliative psychiatric care included establishing palliative care centers, training specialized professionals, combating stigma, and implementing healthcare system regulations.

### Sub-theme 1: Palliative care center

Participants suggested establishing palliative care centers to holistically address psychiatric patients’ needs during condition deterioration.

I wish there were places where patients could directly receive palliative psychiatric care when they are discharged from the hospital or when their condition worsens at home. Unfortunately, this support is not yet available. (Participant 9)

### Sub-theme 2: Training of health professionals

Participants emphasized the need for specialized training for all healthcare professionals to effectively provide palliative psychiatric care.

How should care processes be managed? How should the communication process between the patient and their family be handled in these care processes? These should be addressed separately. Nurses lack sufficient knowledge on each subject. We need to improve ourselves. Training should be provided to increase nurses’ competencies. Additionally, other healthcare professionals should also be trained. (Participant 12)

For example, specialized psychiatric nurses could work as mentors alongside the psychiatric nursing team. (Participant 3)

### Sub-theme 3: Combating stigma

Participants emphasized the importance of combating the stigma of psychiatric illnesses in order to provide palliative psychiatric care. In this sense, they stated that both health professionals and society need to be made aware.

…To integrate patients more into social life, we need to reduce stigmatization in society. (Participant 7)

### Sub-theme 4: Regulation in the healthcare system

Participants made suggestions for the regulation of the health system in order to provide palliative psychiatric care. They made suggestions for this regulation to start the palliative psychiatric care legislation studies, to have psychiatric nurses work within the Ministry of Health, to increase the resources for psychiatric services and to develop monitoring mechanisms to ensure standardization in inter-institutional care services.

I think the healthcare system is inadequate. Some services seem to be left to individual initiative, and there is no standardization. A good practice implemented in one clinic is not replicated in another. … I believe these should be audited. (Participant 7)

I think having a cadre of specialist nurses within the Ministry of Health would be an important step forward. If nurses who have gained specialization work in their respective fields, the number of qualified personnel in patient care would increase. (Participant 2)

## Discussion

This study examined psychiatric nurses’ views on palliative psychiatric care under 3 themes. Many participants encountered the concept for the first time, defining its aim as addressing physical and psychosocial needs during acute and chronic phases. Although holistic care was mentioned, participants mostly focused on physical care and medication management, neglecting quality of life. When the study data are evaluated in this context, it is seen that the sub-theme of drug-focused care and treatment paradigm, which is part of the theme of barriers to palliative care, shows that both employees and administrators do not prioritize care, instead focusing on treatment and neglecting quality of life, thus supporting the perception of palliative care summarized in the first theme. However, modern palliative care focuses on improving quality of life and extending life expectancy (Chan et al. 2023). In their article on the concept of palliative psychiatry, Westermair et al. (2022) emphasized that an acceptable level of mental health, psychosocial functioning and quality of life cannot be achieved even with the best treatment management in treatment-focused approaches and instead recommended palliative psychiatric interventions that aim to directly reduce harm, relieve suffering and improve quality of life (Westermair et al. 2022).

Participants identified those needing palliative psychiatric care as individuals with severe psychiatric disorders, treatment-resistant conditions, comorbidities, and end-of-life care needs. However, the literature suggests that the appropriateness of palliative psychiatric care is not limited to specific diagnoses and should be provided based on need rather than prognosis (Trachsel et al. 2016; Lindblad et al. 2019; Radbruch et al. 2020). Participants’ association of patients with end-of-life care needs as being within the target group of palliative care reflects a common misconception about palliative care. A review of the literature reveals that half of the psychiatrists in India and two-thirds of psychiatrists in Switzerland associate the term “palliative” directly with end-of-life care (Trachsel et al. 2019; Saber et al. 2022; Stoll et al. 2022). Palliative care applies not only to terminal conditions but also to early-stage and chronic non-fatal conditions for which there is no effective treatment (Radbruch et al. 2020). Therefore, raising awareness of this broader paradigm among psychiatric professionals is essential.

Participants emphasized that they felt inadequate in addressing the physical care of patients, especially those with comorbid conditions or physiological problems due to aging and comorbidity. They noted a need for knowledge and skills related to physical care in cases of comorbidities. The literature contains data on only a local problem area of this problem (Elie et al. 2018; Decorte et al. 2020). In a study conducted in psychiatric clinics by Elie et al. (2018), physical care was identified as the most challenging area (Elie et al. 2018). Similarly, Decorte et al. (2020) highlighted the need for sufficient knowledge and experience in physical care (Decorte et al. 2020). Behavioral problems, impairments in decision-making abilities (Picot et al. 2015; Elie et al. 2018), and communication difficulties (Aurora and Miguel 2023) in individuals with psychiatric disorders pose significant challenges for healthcare professionals in managing physical care. In psychiatric services focused on drug treatment, patients’ physical care needs cannot be met and their psychosocial problems cannot be addressed. In a study examining nurses’ perspectives on palliative psychiatric care, nurses stated that they had difficulty providing psychosocial care to patients. Nurses stated that they had difficulty repairing patients’ distorted relationships with their families, that disease symptoms prevented the patient’s end-of-life wishes from being understood, and that patients had difficulty communicating in the process of coping with fear of death or feelings such as restlessness, denial, and anger (Evenblij et al. 2016).

The findings regarding the participants’ perception of psychiatric palliative care show that psychiatric palliative care is evaluated in a very limited framework focused on the just patient. The palliative care paradigm is a comprehensive approach that is integrated with therapeutic care, encompassing personalized, advanced practices (Den Boer et al. 2019; Decorte et al. 2020; Etgen 2020). It commences from the diagnosis stage and addresses death, the post-mortem process, and the family’s mourning process (Trachsel et al. 2019). It is vital that psychiatric nurses are aware of, and adopt, comprehensive palliative care services, since these have been shown to significantly benefit patients, their families, health professionals, organizations, and health systems (Foti 2009; Trachsel et al. 2016). The psychiatric nurses participating in this study have indicated that psychiatric services with a primary focus on drug treatment have not adequately addressed the physical care requirements of patients. Furthermore, there has been an absence of recognition and management of the psychosocial problems experienced by the patient and their family. These findings underscore the necessity for the establishment of comprehensive palliative care services that can address the holistic needs of patients and their families as evidenced in the literature (Gloeckler and Trachsel 2022).

The findings of the study also point to the barriers to the realization of psychiatric palliative care practices as expressed from an insider’s perspective, clarifying the points that need to be tackled in order to access palliative psychiatry practices with high potential to make significant contributions to the lives of psychiatric patients. These barriers are interrelated and reflect a paradigm that is treatment-oriented, lacks a team-based and holistic approach, and is oriented towards acute interventions. When combined with stigmatization and discrimination, these barriers become even stronger. The literature suggests that there are insufficient institutions to provide end-of-life care for psychiatric patients. At the same time, individuals with serious mental illness are at risk of poverty, homelessness, hunger, and violence (Gutwinski et al. 2021). All of these reasons can make it difficult to find safe and appropriate care environments for individuals with progressive mental illness. In Turkey, day and residential care services are provided for individuals with physical, mental and psychological disabilities of all ages (Kılıç and Yılmaz 2018). There are very few studies on how effectively individuals receiving service in these centers receive service (Kılıç and Yılmaz 2018). Psychiatric wards are also inadequate to provide this care, and residential care homes may be reluctant to accept individuals with mental illness due to stigmatization, few professionals, and inadequate training of health professionals (Steves and Williams 2016). It is known that a holistic approach in psychiatric services can improve patients’ prognoses, help prevent the chronicity of diseases (Solmi et al. 2023), and provide a cost-effective intervention (Sun et al. 2023), but it is insufficiently supported by administrators (Sun et al. 2023). Studies have shown that psychiatric patients face disadvantages in accessing health services, and despite the significant burden that mental disorders pose on public health, the necessary financial resources are not allocated to mental health services (Vecchio et al. 2018; Plana-Ripoll et al. 2019; Momen et al. 2022; Chan et al. 2023). Treatment-oriented service approach, lack of knowledge of professionals and administrators about psychiatric palliative care, stigmatization of individuals with mental illness in society and health professionals, and policies are among the barriers to patients’ access to psychiatric palliative care.

To address these barriers, participants recommended allocating sufficient resources to mental health services, reducing healthcare access inequalities, standardizing services across institutions, establishing palliative care centers, training and supporting mental health professionals in palliative care, and combating stigmatization through awareness initiatives targeting both society and healthcare providers.

## Strengths and limitations

Firstly, the participation of the study’s respondents through the Psychiatric Nursing Association may have led to sampling bias. Nurses affiliated with the association are generally more proactive and knowledgeable about specific issues, which could influence their responses. Furthermore, participants interested in advancing in the field of psychiatry are more likely to respond. However, the fact that the nurses participated in the interview independently from their institutions and in an external environment may have led them to feel freer in their answers. At the same time, the fact that we had the opportunity to reach nurses from 11 different institutions constitutes the strength of the study.

## Conclusion

This study revealed that psychiatric nurses were unfamiliar with palliative psychiatric care, associating it with holistic and individualized care. The literature suggests that the appropriateness of psychiatric palliative care is not limited to specific diagnoses and should be based on need rather than prognosis, but participants indicated that the target group for palliative psychiatric care is severe psychiatric disorders, treatment-resistant conditions, comorbidities and end-of-life care. Another finding of the study is that palliative psychiatric care is not provided due to the current health system’s adoption of a drug-based care and treatment paradigm. It is seen that there is a need to reorganize the health system and establish palliative care centers, train health professionals specialized in this field and efforts to combat stigma.

## Supporting information

10.1017/S1478951526101679.sm001 Erdeniz Güreş and Atlı Özbaş supplementary material Erdeniz Güreş and Atlı Özbaş supplementary material

## Supplementary material

The supplementary material for this article can be found at https://doi.org/10.1017/S1478951526101679.
